# Intracoronary Imaging: Blind to Color and Sex but Not Income or Insurance

**DOI:** 10.1016/j.jscai.2024.102018

**Published:** 2024-05-11

**Authors:** Neelima Katukuri, James C. Blankenship

**Affiliations:** a Department of Cardiology, Orlando VA Medical Center, Orlando, Florida; b Division of Cardiology, University of New Mexico Health Sciences Center, Albuquerque, New Mexico

**Keywords:** intravascular ultrasound, optical coherence tomography, outcomes, percutaneous coronary intervention

It is fashionable to look for sex- and race-based disparities in everything from cardiology journal editorial boards^1^ to cardiac complications of COVID-19.^2^ In that vein, in this issue of *JSCAI*, Ismayl et al^3^ report on disparities in sex, race/ethnicity, and economics in utilization and outcomes of intracoronary imaging. The authors used the National Inpatient Sample database to analyze 2,212,595 weighted hospitalizations with percutaneous coronary intervention (PCI) from 2016 to 2020, of which 204,735 included intracoronary imaging with intravascular ultrasound or optical coherence tomography. They looked at associations of sex, race/ethnicity, and economic categories with use of intracoronary imaging and outcomes of imaging-guided PCI. Sex categories were limited to male or female. Race and ethnicity were limited by the National Inpatient Sample data to considering Black, Hispanic, non-Hispanic White, and “other,” which included Asian/Pacific Islander, Native American, and other. Economic status was categorized according to the average income of the patient’s home ZIP code divided into lowest quartile, middle 2 quartiles, and highest quartile.

Why would one expect to find sex/racial/ethnic/economic disparities in use of intracoronary imaging? This is because such differences have been identified in rates of catheterization and PCI after acute myocardial infarction (AMI),^4^ use of drug-eluting stents in PCI,^5^ referral to cardiac rehabilitation after AMI,^6^ guideline-directed cardiac medical therapy,^7^ access to transcatheter aortic valve replacement,^8^ and just about every other area of interventional cardiology.^9^ It would not be surprising to find differences in use and outcomes of intracoronary imaging as well.

Implicit in the study by Ismayl et al^3^ are the safe assumptions that intracoronary imaging improves PCI outcomes, decreases mortality, reduces target vessel revascularization,^10^ and is underutilized in the United States.^11^ Thus, higher rates of intracoronary imaging in the United States would be expected to further improve stenting outcomes; however, interventional cardiologists know that intracoronary imaging is costly and time-consuming,^12^ which may account for why it is underutilized and why disparities in its use may exist.

The authors’ findings are at once reassuring and distressing. They are reassuring in that logistic regression analysis did not find any independent association between sex and use of imaging nor any association between race/ethnicity and use of imaging. This contrasts with the studies cited above that have shown disparities in access to many cardiac therapies. In the 4-level model of racial discrimination,^13^ the authors’ findings suggest that neither *intrapersonal discrimination* (ie, internalization of other's biases) nor *interpersonal discrimination* (ie, discrimination against individuals by a health care provider) affects the use of intracoronary imaging.

The findings are distressing in that they correlate use of intracoronary imaging with economic status. The patients from the highest income ZIP code quartile were more likely to undergo intracoronary imaging (12.5%) than patients in the lowest quartile (9.2%) or the middle 2 quartiles (10.0%; *P* < .01). Patients with Medicaid insurance were less likely to undergo intracoronary imaging compared with those with Medicare, private insurance, or “self-pay” (adjusted odds ratio, 0.93; *P* < .01). As the authors point out, if economic status affects access to potentially life-saving care such as use of intravascular ultrasound, it would be a valid goal to eliminate such economic barriers.

The authors do not speculate on why income level or insurance status would affect use of intracoronary imaging. As noted above, economic status and insurance status may affect whether one gets into the catheterization laboratory; however, once there, it is hard to imagine operators would ration their use of equipment based on the patient’s economic or insurance status. The explanation for why lower-income patients get less intracoronary imaging may lie at the third level (intra-institutional) or the fourth level (health care system) of the 4-level discrimination model. Lower-income patients tend to go to lower quality, lower-resourced hospitals,^14^, ^15^, ^16^ which may discourage use of intracoronary imaging due to cost. In fact, use of intracoronary imaging varies widely across hospitals, and in 1 study,^12^ hospital characteristics were a much stronger correlate of use of intracoronary imaging than lesion or patient characteristics. Hospital-level or health care system policy-level interventions that encourage use of intracoronary imaging at all hospitals may be necessary to remove economic disparities in intracoronary imaging.

A larger question may be whether the differences observed among patients from different economic ZIP codes in this study are clinically important. Does a 9.2% vs 12.5% use of intracoronary imaging, from lowest to highest economic quartile, make a clinical difference in outcomes? Mortality was 2.5% in the intracoronary imaging patients and 2.9% among those without intracoronary imaging, so increasing the use of intracoronary imaging from 9% to 12% might save about 1 life per 1000 patients undergoing PCI. Perhaps a more impactful strategy would be to raise the rates of intracoronary imaging for all sex/race/ethnicity/income levels in the United States. That would require a larger jump in rates of imaging and produce more benefits than would eliminating disparities in imaging among economic groups. A report from the New York State PCI database^10^ indicated that 22% of PCI involved complex lesions, a subset for which the 2021 American College of Cardiology/American Heart Association revascularization guidelines give a class 2a recommendation for use of intracoronary imaging.^17^ Intracoronary imaging is reportedly used in 85% of cases in Japan.^18^ Hence, although the ideal rate of use of intracoronary imaging may be anywhere from 22% to 85%, it is certainly higher than the 9.2% reported in this study.

With respect to outcomes, the authors report that among patients *with* intracoronary imaging, there were no statistically significant differences in adjusted mortality rates among different sex, race, ethnicity, or economic groups. Significant differences were observed among racial/ethnicity groups in patients *without* intracoronary imaging, although this may just be the result of achieving statistical significance with a larger number of patients. Significant excesses in mortality for middle- and low-income patients (adjusted odds ratios, 1.06-1.18; *P* = .01; compared with high income patients) were observed in the group *without* intracoronary imaging, perhaps due to their living in poorer economic ZIP codes with lower resourced, lower quality hospitals.

In summary, it is good news that sex, racial, and ethnic disparities reported in other areas of interventional cardiology are not found in this study. The economic disparities suggest room for improvement. Returning to the 4-level discrimination model, perhaps a solution at a system level is better reimbursement for lower-resourced hospitals and universal health insurance for all citizens that support higher rates of intravascular imaging across the spectrum of PCI cases.
